# Missed diagnosis in first trimester with ruptured tubal ectopic pregnancy in second trimester: a case report

**DOI:** 10.1097/RC9.0000000000000058

**Published:** 2026-01-09

**Authors:** Sonam Dechen, Nidup Gyeltshen

**Affiliations:** a Faculty of Postgraduate Medicine, Khesar Gyalpo University of Medical Sciences of Bhutan, Thimphu, Bhutan; b Department of Obstetrics and Gynecology, Jigme Dorji Wangchuck National Referral Hospital, Thimphu, Bhutan

**Keywords:** bicornuate uterus, case report, ectopic pregnancy, emergency laparotomy, ruptured ectopic pregnancy, salpingectomy, tubal pregnancy

## Abstract

**Introduction and importance:**

A ruptured tubal ectopic pregnancy in the second trimester is a rare presentation. In a reproductive age group presenting with a history of amenorrhea, per vaginal bleeding, abdominal pain with an empty uterus should always be suspected for ectopic pregnancy. Herein, we report a case of a ruptured tubal ectopic pregnancy in the second trimester, which was diagnosed during laparotomy for a suspected ruptured bicornuate uterus.

**Case presentation:**

A 29-year-old, married, with past history of left salpingectomy, second presentation to a district hospital in Bhutan was referred twice for suspected ectopic pregnancy, misdiagnosed as pregnancy in a bicornuate uterus on ultrasonography initially.

**Clinical discussion:**

During the second referral, an emergency laparotomy found a ruptured right tubal ampullary ectopic pregnancy at 13 weeks 4 days period of gestation, massive hemoperitoneum and bowel adhesion in the left adnexal region. She had an uneventful postoperative recovery.

**Conclusion:**

Presentation of ectopic pregnancy in the late first trimester is rare and leads to increased maternal morbidity and mortality. While there are evidences of a bicornuate uterus with an intrauterine pregnancy being mistaken for an ectopic pregnancy, the reverse can also be true, as in our case.

## Introduction

Ectopic pregnancy is the growth of the implanted fertilized ovum outside of the uterus^[1]^. The incidence of ectopic pregnancy in the national referral hospital in Thimphu was 12.7/1000 pregnancies^[2]^. It presents with a classical triad of amenorrhea/positive urine pregnancy test, per vaginal bleeding, and abdominal pain^[3]^. Risk factors for ectopic pregnancy include pelvic inflammatory disease, smoking, history of prior ectopic pregnancy, history of fallopian tube surgery, and assisted reproduction techniques for fertility^[3]^. Ectopic pregnancy is diagnosed with serum beta human chorionic gonadotropin (β-hCG) levels and confirmed through transvaginal ultrasonography (TVS)^[4]^. Most ectopic pregnancy occurs in the fallopian tube; 70% ampullary, 12% isthmic, 11.1% fimbrial, and 12.4% interstitial^[5]^. Management of ectopic pregnancy is based on the viability of pregnancy, gestational age, maternal vital stability, desire for future pregnancy, and the skills of the obstetricians and gynecologists. Ld Aliyu *et al* reported a case whereby a bicornuate uterus mimicking ectopic pregnancy was found to have a bicornuate uterus with an intact pregnancy^[6]^. Here we present a case of ruptured tubal ectopic pregnancy at 13 weeks 4 days period of gestation, initially diagnosed as pregnancy with a bicornuate uterus. The information on this case report was secured by the retrospective review of the patient’s medical documents. The case had been reported in line with the SCARE 2025 criteria^[7]^. Written informed consent was obtained from the patient to publish her de-identified clinical details in this case report.

**HIGHLIGHTS**

- Presentation of ectopic pregnancy in the late first trimester is rare.
- Presentation of ectopic pregnancy in the late first trimester leads to increased maternal morbidity and mortality.
- While there are evidences of a bicornuate uterus with an intrauterine pregnancy being mistaken for an ectopic pregnancy, the reverse can also be true, as in our case.

## Case presentation

### Patient information

**Figure 1.**
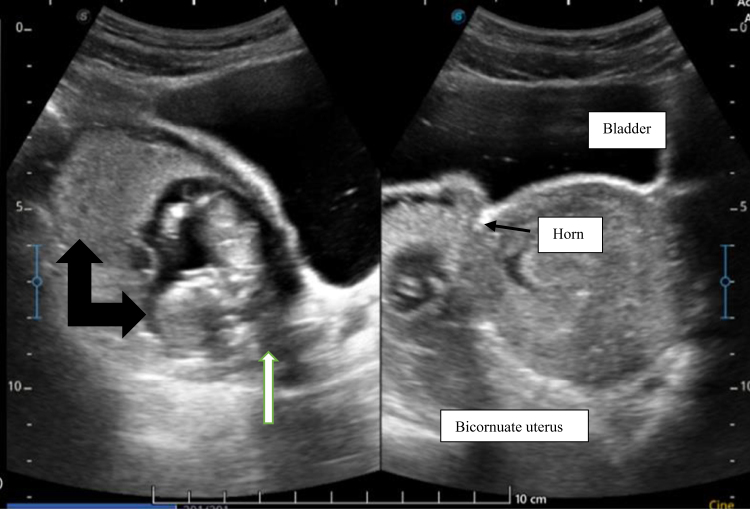
Longitudinal image of the gestational sac showing the placenta and the fetus by the white arrow. The cervix is labelled by the white arrow.

**Figure 2.**
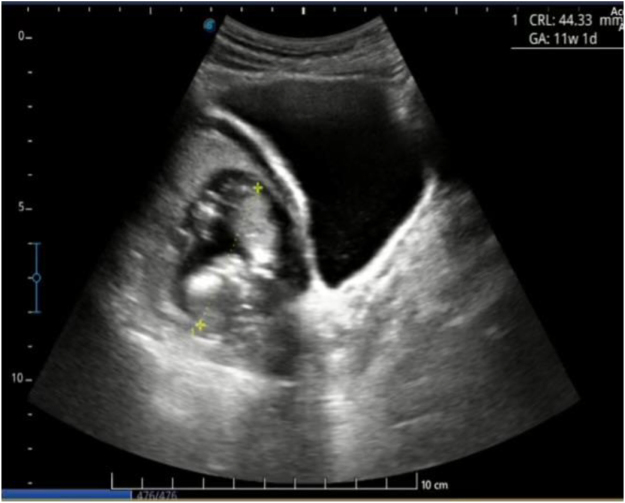
Longitudinal image of the gestational sac, with CRL measured as 11 weeks 1 day period of gestation.

A 29-year-old nulliparous woman presented to a district hospital in Bhutan on 31 December 2024 with a history of amenorrhea of 3 months duration. During investigation, her serum β-hCG was 28 032 mIU/ml. A transabdominal ultrasound was done, which showed a live embryo with a crown-rump length (CRL) measuring 4.2 cm, corresponding to 11 weeks 1 day, in the right adnexa with fetal movement. For further management, she was referred to the national referral hospital on the same day. The patient was stable on admission, and the findings of abdominal examination were insignificant. Ultrasound done in the emergency department (Figs 1 and 2) was suggestive of a bicornuate uterus with an intrauterine pregnancy.

She was admitted to the ward and further evaluation with transvaginal sonography on 14 December 2024, showed a bulky uterus which was anteverted with fetus of CRL 5 mm with cardiac activity in the right adnexa. No free fluid was noted. On the same day for confirmation, an ultrasound scan was repeated by a maternal-fetal medicine (MFM) specialist and the findings were suggestive of a bicornuate uterus with a live intrauterine corresponding to 11 weeks and 2 days. Ovaries were normal, and minimal free fluid was noted in the pouch of Douglas.

She was monitored for 2 days, and since she was asymptomatic, she was discharged and advised to continue pregnancy with MFM review at 18–20 weeks period of gestation.

She presented to a district hospital with acute abdominal pain on 31 December 2024 at 0700 h, which was 18 days after she was diagnosed with a bicornuate uterus with a live intrauterine pregnancy.

### Social and medical history

She had been married for the past 2 years and had a good family support. She gave a history of laparotomy right salpingectomy being done in the district hospital in the year 2022 for unruptured left ectopic pregnancy at 12 weeks. She gave a history of using injection depot medroxyprogesterone acetate as the method of contraception, which is commonly used in many of healthcare facilities in Bhutan. However, she gave a history of not using any sort of contraception for the past 7 months.

### Investigation and management

At the district hospital, she was found to be conscious, afebrile, oriented to time, place and person. She was looking ill, pallor, hypotensive with blood pressure 90/60 mmHg with pulse rate of 82 bpm. She was airlifted as a case of rupture of the uterus. An air ambulance has been made available in Bhutan since November 2015.

At the emergency department of the national referral hospital, she was suspected of ruptured cornual pregnancy at 13 weeks 4 days period of gestation in shock. The findings were recorded and discussed with patient’s party regarding the need for laparotomy, and the couple agreed to the same. Informed written consent for the surgery was taken, and preoperatively, preparation was done with blood reservation. Laparotomy with right salpingectomy by the on-call obstetrician team.

**Figure 3.**
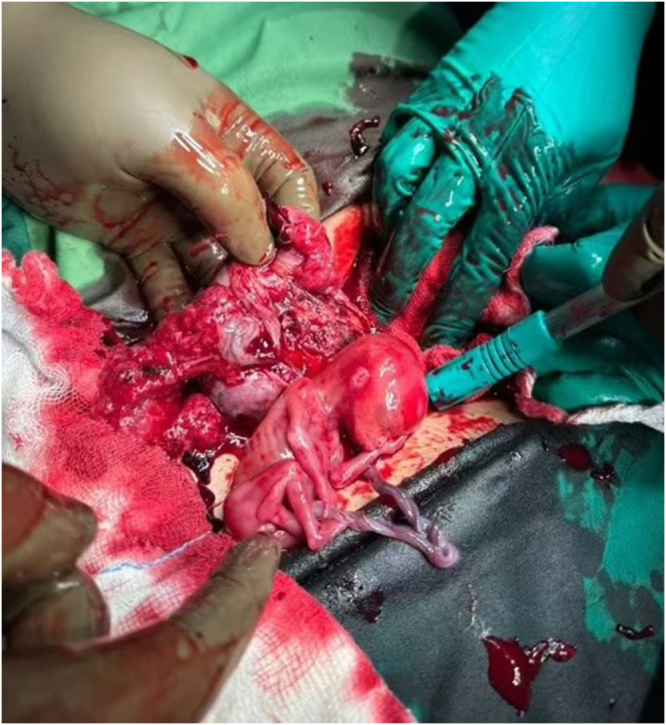
Ruptured right tubal ampullary ectopic pregnancy in a 29-year-old lady.

**Figure 4.**
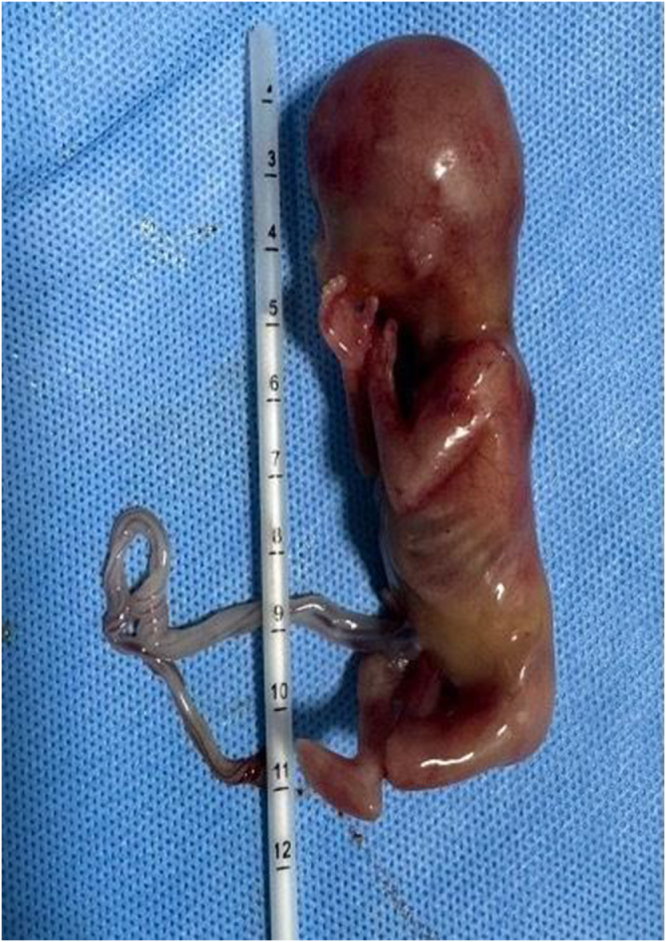
13 weeks 4 days fetus with umbilical cord.

Intraoperative findings documented (Figs 3 and 4): (1) 2500 ml hemoperitoneum, (2) fetus seen in the abdomen, (3) ruptured right tubal ampullary ectopic pregnancy, (4) left tube not seen status post left salpingectomy in the past, and (5) bowel adhesion in the left adnexal region. Two units of packed red blood cells were transfused intraoperatively.

She was counselled regarding the intraoperative findings by the senior obstetrician and gynecologist consultant on postoperative day 1 and was monitored till postoperative day 3. Postoperatively it was uneventful. Stitches were removed on postoperative day 7. Histopathological examination confirmed the intra-operative diagnosis of a ruptured right tubal ectopic pregnancy.

**Figure 5.**
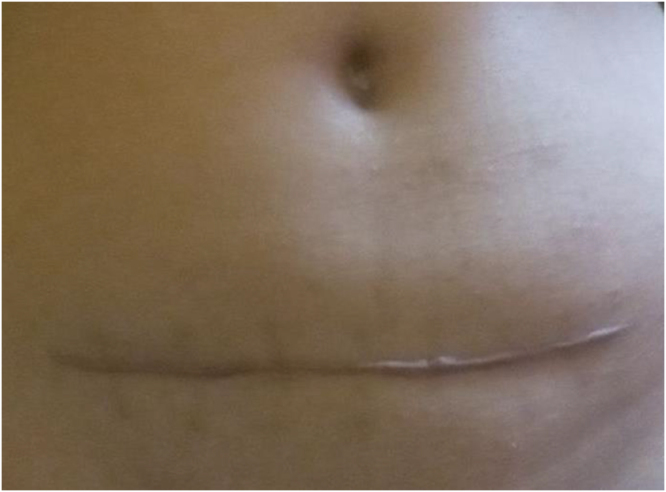
A Low transverse incision in a 29-year-old lady to remove ruptured tubal ampullary ectopic pregnancy at 13 weeks 4 days period of gestation.

On review 6 weeks after the surgery, the wound was healthy (Fig. 5), and she was found to be attending her daily activities.

## Discussion

In this case, we present a case of ruptured tubal ectopic pregnancy at 13 weeks 4 days period of gestation after being misdiagnosed as an intrauterine pregnancy in a bicornuate uterus. Ectopic pregnancy is the implantation of the fertilized ovum outside of the uterus^[1]^. A study conducted by Sonam Gyamtsho *et al*^[2]^ found that the incidence of ectopic pregnancies was 12.7/1000 pregnancies in the national referral hospital, Thimphu, Bhutan.

The commonest site being within the fallopian tube (90%), followed by ampulla (70%), isthmus (12%), and fimbria (11%)^[2,8]^. Many risk factors are associated with ectopic pregnancies, such as pelvic inflammatory disease, smoking, history of prior ectopic pregnancy, history of fallopian tube surgery, and assisted reproduction techniques for fertility^[3]^. In our case, the patient had a history of PID and a history of prior ectopic pregnancy. The commonest form of presentation for ectopic pregnancy; abdominal pain, amenorrhea, and history of per vaginal bleeding. In our case, she had presented with amenorrhea and abdominal pain, following which she was suspected of ectopic pregnancy, which was later misdiagnosed as she presented in the late first trimester.

It is rare for an ectopic pregnancy to persist beyond the first trimester, but it has been reported by many^[9-11]^. Interstitial ectopic pregnancy is the commonest ectopic pregnancy mistaken as the intrauterine pregnancy. And it is found to be rare for the fallopian tube to dilate and accommodate pregnancy beyond the first trimester. It is a diagnostic challenge to diagnose through ultrasonography only and requires further follow-up for better outcomes.

Ectopic pregnancy was diagnosed by β-hCG level and TVS^[4]^. Tubal ectopic pregnancy is often diagnosed through ultrasonography but it is often missed in advanced gestational age^[5]^. Patient presenting with acute abdominal pain in the second trimester of pregnancy, suspicion of tubal pregnancy should be kept as a possible differential diagnosis. Whereas in our case, since she had presented at a late first trimester, the transabdominal ultrasonography impression was a bicornuate uterus with pregnancy. Since the patient improved clinically in the first admission, ectopic pregnancy was ruled out. It was a diagnostic challenge to detect tubal pregnancy in the second trimester, and a possible diagnosis of tubal pregnancy was missed.

Based on the hemodynamic stability, laboratory findings of β-hCG level, complete blood counts and desire for fertility in the future, ectopic pregnancy can be managed by conservative, medical and surgical modalities^[4,12]^. In our case, since the patient presented in shock, there was no role of conservative management. She had undergone emergency laparotomy with right salpingectomy, which confirmed the diagnosis of ruptured tubal pregnancy, similar to a case series by Sachan *et al*^[13]^. The case series reported a case of viable tubal ampullary ectopic pregnancy at 14 weeks of gestation with a copper T *in situ* and a second case of advanced gestational age with failed induction of labor, upon laparotomy diagnosis of ectopic pregnancy was made.

To detect early ectopic pregnancy and prevent late first-trimester rupture, it is essential to obtain a proper history, especially regarding amenorrhea, abdominal pain, or per vaginal bleeding/spotting, particularly when risk factors exist, such as previous ectopic pregnancy, tubal surgery, smoking, intrauterine device *in situ*, and pelvis inflammatory diseases^[14,15]^. If laboratory facilities allow measurement of serial β-hCG, clinicians should observe for a plateauing or suboptimal rise. When the β-hCG level is above the discriminatory zone (1500–3000 IU/l), and there is no intrauterine pregnancy seen on ultrasound, ectopic pregnancy is highly likely^[14,16]^. At 5–6 weeks of gestation, TVS plays a crucial role in helping in early detection, thereby preventing late first-trimester rupture^[15]^. A combination of serial β-hCG follow-up, repeat TVS and vigilant monitoring for increasing abdominal pain, shoulder tip pain, per vaginal bleeding significantly reduced the risk of delayed presentation with rupture.

Recent reports have highlighted uncommon sites of ectopic pregnancy. Pham *et al* described rare cases of implantation of pregnancy on the pelvic sidewall muscles/peritoneum and the live, both extremely unusual forms of abdominal ectopic pregnancy sites^[17]^. Broad ligament ectopic pregnancy is another rare entity, where implantation occurs between the two layers of the broad ligament, which mimics adnexal pathology until confirmed intraoperatively^[18]^. Reviews on early and atypical presentations also note that several non-tubal implantation sites (cesarean scar, cervical interstitial and ovarian ectopic) are associated with a high risk of severe hemorrhage if not promptly diagnosed^[19]^. Recognition of these rare implantation sites is important for timely diagnosis and prevention of life-threatening complications.

## Conclusion

Presentation of ectopic pregnancy in the late first trimester is rare and often leads to increased maternal morbidity and mortality. While there are evidences of a bicornuate uterus with an intrauterine pregnancy being mistaken for an ectopic pregnancy, the reverse can also be true, as in our case.

## Data Availability

Data are available on resonable request.
